# Ultrasonographic Evaluation of Sub-Clinical Synovitis in Juvenile Idiopathic Arthritis: The Disease Classification and Management

**DOI:** 10.3390/life12111750

**Published:** 2022-10-31

**Authors:** Rather Suhaib, Rasool Riaz, Shamas Haris, Bhat Mushtaq, Robbani Irfan, Shah Omair, Khushdil Ajaz, Faheem Shehjar, Hamed A. El-Serehy

**Affiliations:** 1 Department of Radiology, Sher-i-Kashmir Institute of Medical Sciences Soura, Srinagar 190011, India; 2 Department of Internal Medicine, Sher-i-Kashmir Institute of Medical Sciences Soura, Srinagar 190011, India; 3 Department of Paediatrics, Sher-i-Kashmir Institute of Medical Sciences Soura, Srinagar 190011, India; 4 Department of General Surgery, Government Medical College, Srinagar 190001, India; 5 Department of Medicinal and Biological Chemistry, University of Toledo Medical Centre, Toledo, OH 43614, USA; 6 Department of Zoology, College of Science, King Saud University, Riyadh 1451, Saudi Arabia

**Keywords:** ultrasonography, synovitis, pediatric, high-resolution linear probe, Power Doppler

## Abstract

Background: Ultrasonography (USG) is a perfect device for analyzing more than one joint in rather brief intervals of time and is well accepted by children with no harmful ionizing radiation, usually does not require sedation, and can be carried out without difficulty in an outpatient setting. Purpose: To demonstrate the ability of ultrasonography (USG) in detecting clinical and subclinical synovitis in children with juvenile idiopathic arthritis (JIA) and compare the USG findings with clinical findings. Methods: 20 patients with JIA diagnosed according to the ILAR criteria were include. A total of 208 joints were examined both clinically and ultrasonographically for detection of synovitis. The presence of subclinical synovitis detected by USG was sought and its effect on the classification of JIA was assessed. USG assessment was done using the High-Resolution Linear probe including both grey scale and Power Doppler assessment. Results: The mean age of patients was 10.2 years with average disease duration of 5.9 months. A total of 49 joints (23.5%) had clinical synovitis and 59 joints (28.4%) had USG synovitis out of a total of 208 joints. A total of 14 joints had subclinical synovitis (8.8% out of the 159 clinically normal joints) upon USG. USG additionally brought about classifying three patients as having poly articular disorder who had been considered as oligo articular upon clinical examination. Conclusion: USG assessment of subclinical synovitis in JIA patients is an essential component of classifying the disease and detects more joints with synovitis than clinical examination; however, both are complimentary and should be used in combination in all patients with JIA.

## 1. Introduction

Juvenile Idiopathic Arthritis (JIA) is a chronic rheumatic disease and the most common pediatric rheumatic disease leading to disabilities in childhood. Its incidence ranges from 1 to 22 per million [1,2,3]. The classical presentation of JIA is defined as the presence of objective arthritis in one or more joints over a period of 6 weeks in patients younger than 16 years, provided other pediatric arthritis’s has been ruled out [4,5]. The diagnosis of JIA mainly rests on the presentation of the patient with arthritic features combined with a physical examination of the involved joints. Biochemical evaluation plays little if any role in the diagnosis of JIA. When diagnosing JIA, the term “arthritis” is to be taken as the presence of joint swelling excluding bony outgrowths/lesions or inactive synovitis. In the absence of joint swelling, limitation of joint movement with associated tenderness, redness, or heat around the joint can be taken as suggestive of arthritis [6,7]. The classification of arthritis in children is based on the International League of Associations of Rheumatology (ILAR) criteria of 1997 which classifies JIA into polyarticular, oligoarticular, systemic, enthesitis-related (ERA), psoriatic, or undifferentiated [8]. The emergence of high-frequency ultrasound probes with the excellent resolution has revolutionized musculoskeletal ultrasonography. USG has allowed clinicians now to detect soft tissue lesions as well as early erosive changes in the bone. USG allows direct assessment of small joints and is now an established imaging modality in rheumatological disorders. The use of color and Power Doppler helps in assessing the blood flow to the synovium and surrounding soft tissues, thereby helping in grading the activity of the disease [8,9,10,11,12,13,14,15]. Musculoskeletal USG has been a game changer, especially in children wherein it is associated with better compliance, lack of radiation exposure, and no need for any sedation or anesthesia. USG also has the added advantage of dynamic evaluation of joints and being readily available and accessible. Multiple joints can be assessed in the same setting and comparisons can be made between the joints [15,16,17,18,19].

The assessment of JIA patients has traditionally been clinical but with the advent of USG, the term subclinical synovitis has come to the fore. The presence of synovial thickening with the increased flow on Power Doppler USG has been accepted as a feature of joint involvement in JIA. This has greatly changed the way JIA patients are now evaluated, wherein many patients with clinically normal joints have been found to have inflammation based on USG findings. This has not only helped in early diagnosis and assignment of patients to different classes of JIA but also helped initiate early treatment and thereby reduce morbidity in affected children. Although MRI has been previously used to identify subclinical diseases in adults, its use in children has been challenging in view of the need for sedation or anesthesia [19,20,21,22,23].

Our study was therefore aimed at assessing the role of USG in pediatric patients with JIA, particularly with regard to early detection of subclinical synovitis, and comparing the clinical and USG assessment of various joints in patients with JIA.

## 2. Materials and Methods

The study was conducted by the Department of Radiodiagnosis and Imaging in collaboration with the Department of Pediatrics and General Medicine at Sher I Kashmir Institute of Medical Sciences, Srinagar, J and K, India. We included all patients who met the ILAR criteria for JIA, attending the outpatient clinic of the Departments of Pediatrics and Rheumatology. We excluded JIA patients with any associated malignancy, mixed connective tissue disorder, or any history of significant trauma. Informed consent was obtained from all patients, parents, or guardians, as appropriate. 

After obtaining the demographic data, a complete clinical evaluation of the patients was done by a rheumatologist with more than 15 years of experience. The clinical evaluation included the application of ILAR criteria and assigning patients to different ILAR categories based on the number and the type of joints involved. The number of joints assessed in each patient was 10 (5 on each side), based on previous assessments done by Collado et al. [14]. The joints assessed included bilateral knee joints, wrist joints, elbow joints, ankle joints, and bilateral Metacarpo-phalangeal joints. A total of 200 joints were assessed based on the Collado et al. joint assessment and 8 additional joints with clinical involvement were also assessed by USG. All the joints were assessed for the presence or absence of swelling, tenderness or pain on motion, and restricted motion. The involvement of a particular joint in active disease (clinical synovitis) was defined based on the presence of swelling or tenderness/pain on motion and restricted motion. 

Laboratory assessment of all patients was done in the form of Rheumatoid Factor (RF), erythrocyte sedimentation rate (ESR), and C-reactive protein (CRP). 

USG assessment was done on the same day or if the patient was non-compliant on the next day in a cool and serene room by a radiologist with more than 10 years of experience in musculoskeletal ultrasounds. The radiologist was kept blinded of the clinical assessment of the patients. A total of 208 joints were assessed ultrasonographically for the presence or absence of synovial hyperplasia, joint effusion, and Power Doppler (PD) signal. US examination was performed with Logiq P5 (General Electric Medical Systems, Milwaukee, WI, USA), equipped with an 8–15 MHz volumetric probe (4D16L) and linear probe (9L). Synovial hypertrophy was indicated by the hypoechoic area in the joint space which was noncompressible and separate from the fat pad of the joint. Joint effusion on the other hand was taken as an anechoic compressible area around the joint. Power Doppler assessment was done in all cases and any vessel dot was considered positive after excluding artifactual signals. In each joint, synovial hyperplasia and joint effusion were graded as follows: 0 = absent, 1 = mild, 2 = moderate, and 3 = marked. Power Doppler signal was graded as follows: 0 = absent, 1 = presence of single vessel dot, 2 = presence of confluent vessel dots in less than half of the synovial area, and 3 = presence of confluent vessel dots in more than half of the synovial area. The assessment of all the joints was done in both longitudinal as well as transverse planes. An abnormal USG was taken to be suggestive of imaging synovitis. The USG protocol followed by our patients was based on the guidelines put forth by the Outcome Measures in Rheumatology Clinical Trials.

Statistical methods:

The data was collected and evaluated using SPSS 28.0.1.1 USA. Descriptive data were analyzed by frequencies and categorical data by percentages and continuous variables by means and standard deviations. Continuous variables were compared using Student’s *t* test. The detection of joint synovitis by clinical and USG means was compared and for all comparisons, a *p*-Value of <0.05 was considered statistically significant.

## 3. Results

### 3.1. Patient Characteristics

A total of 20 patients, 13 boys and 7 girls were included in the study. The mean age at disease detection was 10.2 years (range 3–14 years) (Table 1). 

### 3.2. Disease Presentation and Duration

Most of our patients (*n* = 14, (70%)) presented with joint pain whereas the others had joint swelling (*n* = 6 (30%)) and restricted movement (*n* = 5 (25%)). The average duration of symptoms in our patients was 5 months.

### 3.3. Laboratory Parameters

We found 16 patients who had positive lab findings in the form of abnormal ESR (*n* = 7.35%), CRP (*n* = 7.35%), and a positive RF (*n* = 2.10%). Four (20%) of our patients had normal laboratory findings.

### 3.4. Clinical Findings

In total, 208 joints were assessed both clinically and with USG. Upon clinical examination, 25 joints (12%) were swollen, 35 joints (16.8%) were tender, 26 joints (12.5%) had restricted motion, and 49 joints (23.5%) had active disease (i.e., had clinical synovitis). Among the 49 joints with clinical synovitis, the most frequently affected were the knees (*n* = 25, (51%)), followed by the ankles (*n* = 6, (12%)), elbows (*n* = 4, (8%)), wrists (*n* = 3 6%), and 2nd MCP (*n* = 3 6%). Eight joints (16%) that were not a part of the original protocol were included because of their clinical involvement and included 5 (10%) Hip joints, 1 (2%) 3rd MCP, and 2 (4%) Metatarso-phalangeal joints.

### 3.5. USG Findings

**Figure 1:**
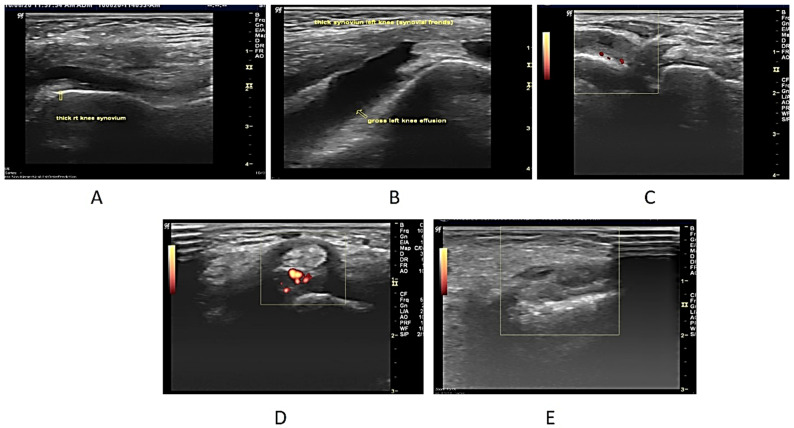
(**A**). Longitudinal USG image of the right knee in a 7-year-old male patient who presented with bilateral knee swelling and mild fever. ESR was also raised. On ultrasonography, both knees revealed synovial hypertrophy with mild effusion in the right knee joint (arrow). (**B**) Longitudinal USG in a 12-year-old girl child presenting clinically as a case of multiple joint pain and swelling in both knees. On examination, both knees were swollen. ESR and CRP were raised. The ultrasonographic image reveals Joint effusion with Synovial hyperplasia confirming clinical findings. (**C**) USG image in a 6-year-old Male child with clinically diagnosed JIA having active findings of joint tenderness and elevated local temperature at 2nd MCP. The image reveals synovial hyperplasia with increased vascularity in the thickened synovium on the Power Doppler (Pulse repetition frequency (PRF), which indicated the number of ultrasound pulses emitted by the transducer over a designated period of time of 0.7 kHz. (**D**) Transverse USG image of the posterior tibial tendon in a 10-year-old male patient of JIA showing peri-tendon synovial thickening and increased vascularity suggestive of tenosynovitis. (**E**) USG image at the level of the wrist joint in a 10-year-old male child diagnosed with JIA with no evidence of wrist joint involvement clinically showing evidence of synovial hyperplasia (arrow). Power Doppler showed a lack of flow.

Upon USG evaluation, 47 joints (22.5%) had synovial hyperplasia, 49 joints (23.5%) had joint effusion, and 19 joints (9.1%) had PD signals. A total of 59 joints (10.0%) had USG synovitis (1 or more of the 3 USG abnormalities). USG abnormalities were seen most frequently in the knees, followed by the ankles, elbows, and wrists. A positive Power Doppler was seen in 19 (26%) of the 59 joints with USG synovitis. Most of these patients (*n* = 16) had grade 2–3 PD signal abnormality and all of them had pain clinically. Only 3 patients had grade 1 PD abnormal signals all three had subclinical synovitis (Figure 1A–E).

### 3.6. Joint Distribution

The distribution of joint involvement both clinically as well as on USG and their comparison has been highlighted in Table 2. A relatively higher percentage of patients with USG synovitis but the clinically asymptomatic joint was seen for knee and 2nd MCP. The joints for which there was a greater frequency of clinical synovitis with a negative USG assessment were the ankle (3) and knee (1).

### 3.7. Additive Value of USG

USG documented synovitis in 45 (92%) of 49 clinically synovial joints. Overall, 159 of the 208 joints scanned were clinically asymptomatic. Of these 159 clinically normal joints, 14 (9%) had evidence of subclinical synovitis based on USG findings. Based on these findings, we found USG to have a sensitivity of 97%, specificity of 100%, and overall accuracy of 98%. USG also led to classifying three (15%) patients as having polyarthritis who were classified as having oligoarthritis or who had no synovitis on clinical evaluation.

## 4. Discussion

Our study was carried out over a period of 2 years, including a total of 20 pediatric patients with JIA. We had a male predominance in our study with 65% boys. Although this finding is contrary to the previous studies done by Cattalini M et al. [23] whose study found a female predominance. The difference is probably because of the small sample of patients and lack of dedicated pediatric rheumatology centers in our part of the world, leading to underdiagnosis and treatment of JIA patients by orthopaedicians without a proper diagnosis. The mean age at which the disease was detected was 10.2 years, which is somewhat higher than the previous studies, such as the one conducted by Ahmed S et al. [24] in which the mean age at diagnosis was 7.6 years. The difference again is probably because of the lack of a robust peripheral mechanism that can help early diagnosis of these patients.

The clinical evaluation of our patients revealed that the most common presenting feature includes joint tenderness/pain followed by joint swelling and restriction in the movement of the joint. Most of our patients had been previously treated by local physicians and orthopedics with suspicion of osteomyelitis, which is common in our part of the world. However, it is only after they visited our center that evaluation was done along the lines of JIA. The clinical evaluation was done based on the Collado et al. [18] protocol including 10 joints per patient. The most common clinically involved joint in our study was the knee joint followed by the ankle joint. A total of 49 joints in 20 patients (200 joints) were found to be involved, and an additional 8 joints were evaluated based on clinical involvement. The joint involvement pattern is quite similar to the studies previously conducted by Ahmed S et al. [24]. The predominance of large joints is probably due to their involvement in daily needs and so being instantly picked up and also the difficulty in the clinical assessment of small joints.

Imaging in the form of USG has been recently introduced for the assessment of joint involvement in rheumatological disorders. Its use especially in the pediatric age group has been promising. We evaluated our patients with high-frequency USG to look for features of joint involvement and compare them to the clinical findings. USG is ideally suited for multiple joint assessments. It has been suggested that its routine use allows a marked improvement of a clinician’s capability to detect both early and hidden features of synovitis. Previous studies have demonstrated the poor reliability of clinical examination of joints in children with JIA. It is, therefore, important to investigate the correlation between clinical and US assessment of joint synovitis and to establish whether USG may improve the accuracy of the detection of joint inflammation in children with JIA.

We assessed all 208 joints clinically evaluated and found that 59 joints showed evidence of imaging synovitis as opposed to only 49 joints clinically detected. The detection of synovitis was exclusive to USG in 14 joints which were clinically normal indicating sub-clinical synovitis. Among these patients with sub-clinical involvement of joints, USG was able to change the diagnosis from an oligo articular disease to a poly articular disease in three (15%) patients in our study. Subclinical synovitis was more common in knees and small hand joints. The increased detection of joint involvement by USG can serve as an important means of classifying JIA based on the number of joints involved, as it outscores clinical assessment in this regard, especially in areas with a shortage of experienced pediatric rheumatologists. The superiority of USG in joint assessment has been previously demonstrated by Kirsty E. Haslam et al. [25] who found subclinical synovitis in additional 15 joints in six of their patients. Similar findings were also recorded by Kirsty E. Haslam et al. [25], who found 70 additional joints involved in the disease by USG when compared to clinical evaluation. It was also found an additional 86 (5.5%) joints with subclinical synovitis. We, therefore, conclude that there are many joints in patients with JIA that remain hidden from clinical evaluation, and imaging in the form of high-resolution USG is essential to unmask the entire gamut of joint involvement in these patients. The use of USG can even change the classification of JIA and help guide the management of these patients. We therefore suggest a mandatory USG in all patients meeting the criteria of JIA to assess the number of joints involved. Although the number of joints to be assessed is debatable, we feel that the 10 joint assessment is not only feasible and realistic but also accurate as far as the joint involvement pattern in JIA is concerned. However, in our study, eight joints not a part of our original plan were found to be involved clinically. All these joints were already a part of poly articular disease and there was no change in the classification of JIA in these patients. Thus, we believe that the 10 joint imaging assessment protocol is a good way of evaluating JIA patients; however, if the clinical features suggest an additional joint involvement, USG of that particular joint should also be done.

The clinical evaluation proved to be superior in four joints in our study wherein the USG assessment came out to be a false negative. This was mostly seen in the ankle joint. Similar findings were also observed by Magni-Manzoni et al. [19] and Burns et al. [26] who found clinical overdiagnosis of synovitis in certain JIA patients, especially involving the ankle joints. Many explanations have been given regarding this but we believe that the frequent misinterpretation of teno synovitis around the ankle joint as clinical joint synovitis is one of the leading reasons. Furthermore, the difficulty in USG assessment of a complex joint such as the ankle may contribute to this. Overall, we believe that clinical and USG assessments of joints in JIA are complementary to each other, however, USG has a higher detection rate and is able to diagnose clinically negative joints as being positive for synovitis. We suggest that an initial follow-up USG assessment of all patients with JIA should be made mandatory and the classification of JIA vis-à-vis the number of joints involved should be based on USG findings. The use of USG for classifying JIA may also guide the management of these patients with the introduction of new biological agents. The detection of subclinical synovitis may help in early treatment and use of these drugs and may help alter the course of the disease to better outcomes. The use of USG for assessing the response to treatment and detecting remission is also being studied wherein the presence of subclinical synovitis guides further treatment which would otherwise have been missed clinically. Although our study did not assess these two aspects, we believe that these are promising aspects of the use of USG in JIA patients.

Our study had certain limitations. Firstly, the number of patients was wasted; however, in a developing region like ours, where diagnostic evaluation is limited and the primary health care is infantile, this is quite a reasonable sample size. Secondly, we did not objectively quantify the clinical and USG features in order to further solidify the relationship between the two modalities. However, it is merely the presence or absence of synovitis that is presently being used to guide management in these patients, rather than the degree of synovitis. Thirdly, we did not have a long-term post-treatment follow-up to assess USG changes in patients with imaging diagnosed synovitis. We should emphasize that our results do not mean that USG is an alternative to clinical examination. It should be regarded as a tool that complements conventional clinical examination. It should be acknowledged that joints not assessed in the study may be very important for individual patients and should be evaluated periodically as part of clinical care. For instance, hip, and small hand joints are involved in a sizable proportion of patients with JIA and are an important source of long-term damage. Exclusion of assessment of these joints and of tenosynovitis weakens the study results.

## 5. Conclusions

Clinical and USG assessments of patients with JIA are complementary modalities essential for disease evaluation. Subclinical synovitis detected by USG is an important feature that can guide not only the diagnosis of JIA but also its classification and management. USG assessment should be done in all patients of JIA at presentation and during follow-up. Further long-term studies are however required to establish the role of USG in assessing disease remission.

## Figures and Tables

**Table 1:** Showing the age distribution of the patients with JIA in our study.

Age (in Years)	Number (*n*)	Percentage (%)
0–6	5	25
7–12	7	35
13–16	8	40
Total	20	100

**Table 2:** Showing the distribution of involved joints in the patients based on clinical and USG criteria and their comparison.

Joints	Clinical Synovitis	USG Synovitis	*p*-Value
Knees	25	31	<0.05
Ankles	6	8	0.15
Elbows	5	5	-
Wrists	3	3	-
2nd MCP’s	2	4	0.09
Hips	5	5	-
Small joints other than 2nd MCP’s	3	3	-
TOTAL	49	59	<0.05

## Data Availability

Not applicable.
